# Utility of QR codes in biological collections

**DOI:** 10.3897/phytokeys.25.5175

**Published:** 2013-07-17

**Authors:** Mauricio Diazgranados, Vicki A. Funk

**Affiliations:** 1 Department of Botany, National Museum of Natural History, Smithsonian Institution, Washington, DC 20013-7012, United States of America

**Keywords:** Barcode, catalogue, collection, database, hard-linking, matrix code, museum, QR code

## Abstract

The popularity of QR codes for encoding information such as URIs has increased exponentially in step with the technological advances and availability of smartphones, digital tablets, and other electronic devices. We propose using QR codes on specimens in biological collections to facilitate linking vouchers’ electronic information with their associated collections. QR codes can efficiently provide such links for connecting collections, photographs, maps, ecosystem notes, citations, and even GenBank sequences. QR codes have numerous advantages over barcodes, including their small size, superior security mechanisms, increased complexity and quantity of information, and low implementation cost. The scope of this paper is to initiate an academic discussion about using QR codes on specimens in biological collections.

## Introduction

There are 55,000 museums in 202 countries containing a variety of collections from art to zebras (De Gruyter 2012). More than 7,000 biological collections worldwide (CBoL et al. 2013) preserve 1.2–2.1 billion specimens (Ariño 2010), although only ~405 million have been digitized (GBIF 2013). With such a large number of specimens, it is critical that the information be made available for research. However, the best way to link specimens to databases and other materials is still under discussion. Some options include *unique specimen identifiers* (USIs), *globally unique identifiers* (GUIDs), *life science identifiers* (LSIDs).

Currently, one of the most frequently used methods is *Barcoding*. This method was implemented in biological collections in the 1990s at INBio and the Smithsonian Institution (Janzen 1992, Thompson 1994). Barcodes are one-dimensional optical representations, where widths and spacing of parallel lines are translated primarily into numeric data. Electronic devices decode the information (usually a 13-digit number and a few letters), which is linked to a database. The idea for modern barcodes arose in 1948 in response to the need from industry to develop a system to quickly capture product data at supermarkets during the check-out process (Brown 1997). By 1949, Bernard Silver and Norman J. Woodland, both from Drexel University, filed a patent application with the prototypes of barcodes called “Classifying Apparatus and Method”, issued in 1952 (Brown 1997, Woodland et al. 1952). By 1966 the first barcodes started to be used commercially (Brown 1997); in 1973 George J. Laurer proposed a standardized barcode system called Uniform Product Code (UPC), and in 1974 the first UPC scanner was installed in a Marsh supermarket in Troy, Ohio (Brown 1997). A 10-pack of Wrigley’s Juicy Fruit chewing gum, today preserved at the National Museum of American History, was the first product in history to have a barcode on it, and their use in commercial situations increased rapidly.

Soon various sectors demanded smaller codes, capable of storing more information and more character types. Matrix codes, also called two-dimensional (2D) codes, were the ideal solution. Among them, QR codes (abbreviation from Quick Response Codes) have increased in popularity during the last few years. They were originally invented in 1994 by a Toyota subsidiary Denso Wave Incorporated (2013), which has chosen not to exercise their patent rights (Denso Wave Incorporated 2013).

QR codes have nine standard features (Denso Wave Incorporated 2013):

1. Capacity to handle different types of data: numeric and alphabetic characters, Kanji, Hiragana, Katakana, symbols, binary and control codes.
2. Large capacity: up to 7,089 numeric and 4,296 alphanumeric characters can be encoded (hundreds of times more than in a barcode).
3. Small printout size: the same information can be encoded in a QR code one-tenth smaller than a barcode.
4. High speed scan:omni-directionally readable, with position detection patterns circumventing the negative effects of background interference.
5. Universal standardization: AIM International Standard, Japanese Industrial Standard and ISO International Standard (ISO/IEC18004).
6. Dirt and damage error correction: QR codes allow a maximum of 30% of damage without losing information, with four levels of security (L (7% of tolerance), M (15%), Q (25%) and H (30%)).
7. Compartmentalization: QR codes can be divided into multiple data areas (as many as 16), allowing smaller printouts.
8. Flexible representation: shapes and colors of modules can be changed, even allowing for artistic representations (QR code Art).
9. Readability: QR codes can be read by any smartphone, tablet or laptop with a camera, using freely available software.

A few remarkable uses of QR codes go beyond the codes themselves. For instance, websites linked in QR codes can be displayed in the user’s preferred language. This was first implemented by QRpedia (http://qrpedia.org) in 2011, to deliver a Wikipedia article in the user’s language using just one QR code. This is how it works:

1. The QR code has to encode an URL containing the domain name “qrwp.org” and the path (final part) to the title of a Wikipedia article. For example, if the Wikipedia article’s URL in Spanish is http://es.wikipedia.org/wiki/Asteraceae, the QR code should encode the URL http://es.qrwp.org/Asteraceae.
2. When the device navigates to the URL contained in the code, its language setting is sent to the QRpedia web server as well (e.g. English).
3. The QRpedia server then uses Wikipedia’s API to look for a version of the article in the language specified (e.g. English), and if it finds one, returns it in a mobile-friendly format. If none are found, then the QRpedia server offers a choice of the available languages, or a Google translation. In the example above the resolved URL would be http://en.m.wikipedia.org/wiki/Asteraceae.

**Figure 1.**
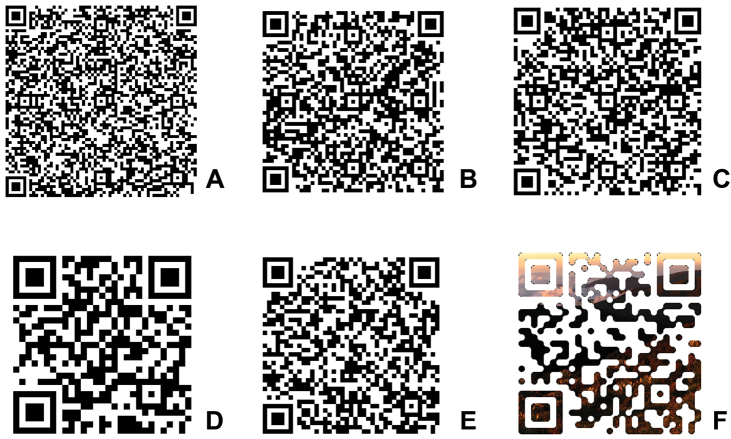
Examples of potential uses of QR codes in Natural History collections. **A** “*Ruilopezia cardonae* (Cuatrec.) Cuatrec. (coll. M.Diazgranados & R.Sánchez 3257). Cited in: Diazgranados M (2012) A nomenclator for the frailejones (Espeletiinae Cuatrec., Asteraceae). PhytoKeys 16: 1–52 (http://www.pensoft.net/journals/phytokeys/article/3186/abstract/)” **B** “Genbank Accession JN837330: http://www.ncbi.nlm.nih.gov/nuccore/JN837330.1” **C** “Photographs of this collection: http://espeletia.org/galleries/main.php?g2_view=tagtree.VirtualAlbum&g2_tags%5B0%5D=815&g2_albumId=7” **D** “http://en.qrwp.org/Sunflower” **E** “Statistics of Fig. 1D: http://qrwp.org/stats.php?path=Sunflower” **F** “http://espeletia.org/”. Readability of these QR codes was tested using an iPhone 4 with Qrafter and Quick Scan.

Another relevant characteristic is the possibility of providing statistics (e.g. with Google Analytics) about usability of each code: how many times a code has been scanned, location of individuals who scanned it, user’s interest (i.e. where code was located), economic utilities associated to a particular code when transactions are generated, etc. For the statistics to be accurate, a unique URL has to be encoded in a QR code, so that the only way to visit the website is by scanning the QR code (e.g. statistics for Fig. 1D can be checked in the website provided in Fig. 1E).

Applications of QR codes range from commercial tracking, transport and entertainment ticketing, visa and passport information, delivering of Wikipedia articles (QRpedia), songs downloading, to encrypted government data (Denso Wave Incorporated 2013). QR codes are also being implemented in libraries in different ways (Ashford 2010). Information that is typically encoded includes vCard contacts, Uniform Resource Identifiers (URI), e-mail addresses, map directions and text.

Despite the fast expansion and popularization of QR codes, they have not been openly incorporated into natural history collections. Their use as USIs or to replace labels in small specimens (e.g. insects) has been briefly suggested in previous works (Blagoderov et al. 2012, Mantle et al. 2012, Schuh 2012), without giving details about advantages or implementation. Currently botanical gardens, a few zoos and various museums are using QR codes to link, for instance, specimens in exhibits to Wikipedia articles.

Some of the reasons to explain their reluctant appearance in natural history collection are:

1. Lack of general knowledge about potential and implementation of QR codes: according to eMarketer (2013), 23–36% of adults (25 to 34 years old) in USA and Europe have scanned a QR code. People usually associate QR codes with URLs, but that is only a limited use.
2. Concerns about the permanence of this technology: collection managers are often unwilling to invest time and resources in a new technology, if they are unsure about its long-term permanence. This is part of the reason there was a lag time of 26 years between the original commercial use of barcodes and their use in natural history collections. But there is also a concern that with technology changing so fast, the long-term permanence of QR codes is difficult to predict.
3. Security concerns (i.e. malicious links or websites). Since QR codes are illegible for the human mind, curators are afraid to link their devices to undesirable websites.
4. Implementation costs: as discussed below, implementing a new technology can be expensive. According to Green (2010), implementing a barcode tracking solution in a middle-size organization costs in average €40,000 (including software licenses, barcode and wireless mobile computer equipment and professional services).

We intend to show the advantages of using QR codes to hard-link specimens to digital resources associated with them. In addition to hundreds of millions of iPods (Apple Inc.) and tablets, there are more than one billion smartphones in use, projected to double by 2017 according to Sui (2013). Virtually any curator or visitor could use one of these devices to quickly access digital information related to the specimen.

QR codes have numerous potential uses in Natural History collections: 1) to provide metadata (e.g., rights of use, proposed citations, projects or particular collections, references, collector contact information); 2) to provide supplementary specimen or species information (e.g., chromosome counts, additional field notes, ecosystem details); 3) to link to digital resources (e.g., photographs, maps, videos, audios, supplementary information); and 4) to provide this information in multiple languages (see examples in Fig. 1). Even though multiple methods have been used in the past to do all this, QR codes provide a unique opportunity to use a personal device (e.g., a smartphone) to perform these tasks in a fast, simple, and graphical way. With QR codes the user would not have to write the collection identifier in a notebook, find a computer, and search in a database, they would only need to point their devices at the code to obtain the links for photographs, maps, etc. When the Internet is accessible through the telephone network (the usual situation in smartphones and tablets), Wi-Fi is not even necessary. Because traditional Barcodes require special scanners plugged to computers with access to the database the information is accessible only by staff. QR codes, however, can contain links that will allow any user to access all the information and some applications allow users to scan multiple QR codes and save results in numerous formats, which would speed up data gathering. Finally, QR codes could be even used to backup digital information from specimens.

## General considerations and recommendations

Projects that implement QR codes on natural history specimens require clear goals. For instance, if Uniform Resource Locators (URLs) are going to be encoded, long-term permanence of URLs must be guaranteed. An alternative for small institutions is the creation of Persistent Uniform Resource Locators (PURLs) that point to other URLs (OCLC 2013). Various PURLs resolvers such as OCLC (2013) are available for free on the Web. Examples of PURLs are the digital object identifiers (DOIs), commonly used in scholarly materials (journal articles, books, etc.). The creation of PURLs is simple and batches of PURLS can be created via API or with Java, Perl or Python codes available online (e.g. Arase 2009, Kaszuba 2013, Perl Training Australia 2013). The resulting URLs should be thumb-interactive, purpose-driven and easy to read using smartphones or any other mobile scanning device.

**Figure 2.**
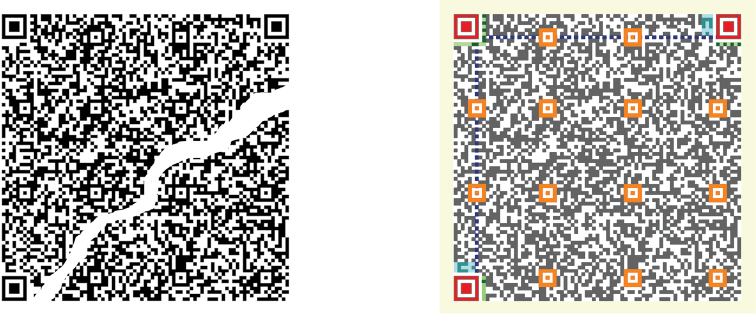
QR code structure and tolerance. Left: QR code with the maximum level of error correction (H: 30% of tolerance to damage). Right: Structure of the same QR code: yellow: quiet zone; red: position; orange: alignment; blue: timing; green: format information; cyan: version; grey and white: data and error correction modules.<br/> Encoded text (including returns):<br/> “US National Herbarium<br/> Montanoa josei V.A. Funk (Asteraceae)<br/> US Sheet No.: 2325539 | Barcode: 00128657<br/> Specimen photographs: http://collections.mnh.si.edu/search/botany/search.php?action=10&irn=10076557&width=495&height=640”

QR codes follow strict standards (Fig. 2). The minimum unit of information is called a module. The number of modules affects size and amount of information, ranging from version 1 (21 × 21 modules) to version 40 (177 × 177 modules). The minimum size of a module is usually established depending on the printer and reader capabilities. The symbol area requires a 4-module wide margin or “quiet (clean) zone” around it (Fig. 2).

Code size and printer resolution are important to generate readable QR codes. The minimum size of the QR code modules depends on printer head density. Higher density improves quality printing, diminishing the effect of paper width and quality, feed speed fluctuations, distortion of axis, blurring, etc. The minimum module size in a Laser printer at 600 dpi (24 dot/mm) assuming a 4-dot/module configuration is 0.17 mm. A thermal printer at 200 dpi (8 dot/mm) and a 4-dot/module configuration prints a module of minimum 0.5 mm of side. Therefore a version 1 QR code (21 × 21 modules) with 30% of error correction (level H) should have a minimum printed size of 1.05 cm per side, leaving a quiet zone of 2 mm around the margins.

Another factor to consider is the scanner resolution. Standard scanners and smartphone cameras have a resolution of 0.25 mm or less. Roughly 90% of these devices read QR codes of 26 × 26 mm, and the latest phones (2011 or newer) have usually macro capabilities allowing them to scan QR codes of 10 × 10 mm.

**Figure 3.**
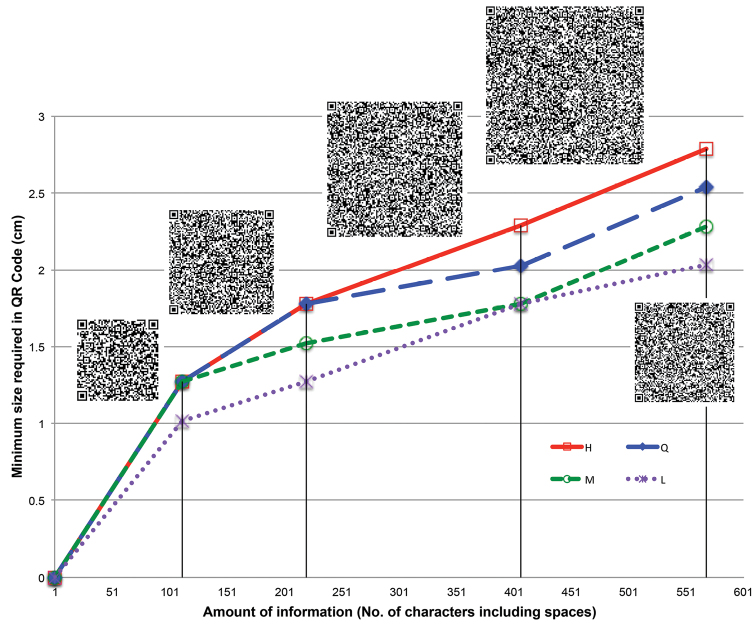
QR code size (side) vs. information for the four levels of error correction (L (purple line): 7% of tolerance; M (green line): 15%; Q (blue line): 25%; and H (red line): 30%). Below the lines QR codes are unreadable by most scanners; above the lines they are all readable.<br/> Encoded text (including returns):<br/> - 111 characters:<br/> “Photographs: http://collections.mnh.si.edu/search/botany/search.php?action=10&irn=10076557&width=495&height=640”<br/> - 219 characters:<br/> “US National Herbarium<br/> Montanoa josei V.A. Funk (Asteraceae)<br/> US Sheet No.: 2325539 | Barcode: 00128657<br/> Specimen photographs: http://collections.mnh.si.edu/search/botany/search.php?action=10&irn=10076557&width=495&height=640”<br/> - 406 characters:<br/> “US National Herbarium<br/> Montanoa josei V.A. Funk (Asteraceae)<br/> Collection: Cuatrecasas, J., Romero Castañeda, R.; 24768; Holotype; 1959-10-10; Colombia; Magdalena; Sierra Nevada de Santa Marta, Hoya del Rio Donachui, Cancurua. Elevation: 2400-2650 m.<br/> US Sheet No.: 2325539 | Barcode: 00128657<br/> Specimen photographs: http://collections.mnh.si.edu/search/botany/search.php?action=10&irn=10076557&width=495&height=640”<br/> - 568 characters:<br/> “Smithsonian Institution<br/> National Museum of Natural History<br/> US National Herbarium<br/> Montanoa josei V.A. Funk<br/> Family: Asteraceae<br/> Collection: Cuatrecasas, J., Romero Castañeda, R.; 24768; Holotype; 1959-10-10; Colombia; Magdalena; Sierra Nevada de Santa Marta, Hoya del Rio Donachui, Cancurua.<br/> Elevation: 2400-2650 m<br/> Verification: Original publication and alleged type specimen examined<br/> US Sheet No.: 2325539<br/> Barcode: 00128657<br/> Specimen photographs: http://collections.mnh.si.edu/search/botany/search.php?action=10&irn=10076557&width=495&height=640<br/> Copyright © Smithsonian Institution”.

To determine minimum size, we tested readability of QR codes with 568, 406, 219 and 111 alphanumeric characters (including spaces), with the four levels of error correction (Fig. 3). All the codes were printed on rough-textured archival paper. Codes were scanned with an iPhone 4® and an iPad 2® using the free version of the software Qrafter (http://keremerkan.net/downloads/). Readable QR codes level H required a minimum size ranging from 1.27 cm (0.5 in) for 111 characters to 2.79 cm (1.1 in) for 568 characters. The minimum size required when using level L ranges from 1.02 cm (0.4 in) for 111 characters to 2.03 cm (0.8 in) for 568 characters. We recommend printing QR codes at least 2 mm bigger than the minimum size readable (e.g. 3 cm/side for codes encoding ~600 characters), and preferably using level H when coding information on specimens of natural history collections. When space is limited (e.g., small labels), lower security codes could be used, and even mini-QR codes.

The cost associated with the implementation of QR codes in biological collections needs to be considered carefully. Programming code for creating QR codes is freely available, and there are numerous QR code generation packages (including an API in Google), most of them free (see Appendix 1). With some free applications it is possible to develop thousands of QR codes for free. However, generating hundreds of thousands or millions of codes would require the purchase of specialized software (prices varying from € $5 to $1,000) or the development of an application using the available programming code. At those scales, even if the price of production is small, the investment in time and resources can be significant.

General considerations and recommendations are summarized in Table 1.

**Table 1.** Important considerations and recommendations for the implementation of QR codes<br/>

**Topic**	**Attribute**	**Considerations & recommendation**
Content	Amount of information	The more text included, the larger the size. A QR code encoding ~600 characters will require a minimum size of ~3cm/side.
	Type of information	QR codes can be use to provide metadata, label information, supplementary information, and links to digital resources.
	Language of content	To deliver content in different languages, a web server such as QRpedia has to identify the language of the scanning device.
	Statistics	URLs encoded in QR codes have to be unique to produce accurate statistics of readability.
	Long-term permanence of URLs	Always use permanent URLs (PURLs)
Design	Size	Size is affected by amount of text, error correction level, paper quality, and printer and scanner resolution. We recommend printing QR codes at least 2 mm bigger than the minimum readable size.
	Error correction	An error correction of 30% (level H) is recommended. This, however, increases the size of the QR code.
	Paper quality	Consider long-term preservation when choosing paper quality. Archival paper has a rough texture, which slightly distorts shapes, affecting the minimum size required for readability. Printing QR codes on labels of new specimens is an inexpensive option. Another more expensive option is using the same materials currently used for Barcodes.
	Cleanness	QR codes require a “quiet” area around them and prefer black/dark print on a white/clear background.
	Printer resolution	Prefer high-quality printers. Test the minimum readable size of QR codes with your printer.
Bulk generation	Generation	Various resources can be used to produce thousands of QR codes for free. The production of batches of hundreds of thousands of QR codes requires adapting available programming code, or purchasing expensive software.
	Cost	The implementation of QR codes in large collections with millions of specimens can be extremely expensive. In those cases we suggest implementing them primarily for the new accessions.
Scanning	Scanner resolution	Most scanning devices (90%) read QR codes of 26 × 26 mm or bigger. For scanning smaller codes macro capabilities are often required.
	Scanning speed	The scanning speed is inversely related to the amount of information in the codes.
	Scanning tips	The device should be kept parallel to the code and as close as possible while still allowing it to focus. Edges of the code must be visible. It requires a few minutes of practice.
Security		QR codes could be used to direct the device to websites with malicious codes. We recommend: 1) collection managers should check QR codes and links from unverified sources before making them available; 2) users should only scan QR codes that have been approved by collection managers; 3) users should deactivate the automatic website launch option in their scanning software so they have the option of declining before it opens.

## Possible utility

QR codes provide a bridge between the digital and the physical world by delivering content to the most used electronic devices. The critical question is, what kind of information would a curator or visiting researcher find useful? QR codes can deliver plain text information combined with multiple links to online content. Plain text information could include institutional information, the label information, rights of use, or additional information not printed on the label, such as agencies and name of the project funding the collecting expeditions. Links to online resources can cover a broad spectrum, from information associated to the specimen itself to data related to the taxonomic entity, locality, research project, etc.

**Figure 4.**
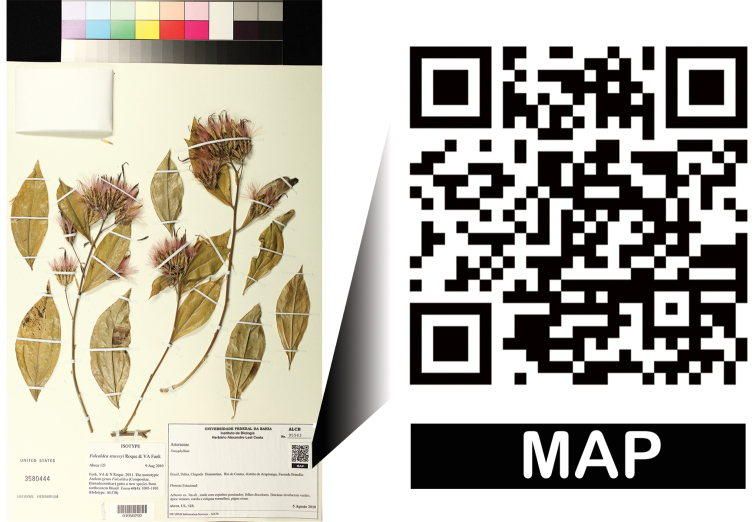
Example of plant specimen at the US herbarium with a QR code linking to a Google map of the collection locality. Encoded text: “http://bit.ly/130tnzO”.

Some examples:

Voucher-additional information (e.g., photographs, illustrations, maps, information in collector’s field books)
Voucher-repositories for duplicates (e.g., Biorepositories: http://www.biorepositories.org/; Index Herbariorum: http://sciweb.nybg.org/science2/IndexHerbariorum.asp)
Notes on conservation status (e.g., species in the IUCN Red List: http://www.iucnredlist.org/; or in CITES: http://www.cites.org/)
Genetic information (e.g., Barcode of Life Database: http://www.barcodinglife.org/; NCBI Genbank: http://www.ncbi.nlm.nih.gov/)
Data (e.g., Dryad: http://datadryad.org/)
Nomenclatural resources, digital keys and databases (e.g., Lucid keys: http://www.lucidcentral.com/; Tropicos: http://www.tropicos.org/; International Plant Names Index: http://www.ipni.org/; Integrated Taxonomic Information System: http://www.itis.gov/; Global Names: http://www.globalnames.org/; The Plant List: http://www.theplantlist.org/)
Checklists, catalogues and encyclopedias (e.g., Catalogue of Life: http://www.catalogueoflife.org/; Encyclopedia of Life: http://eol.org/; Species 2000: http://www.sp2000.org/; eFloras: http://www.efloras.org/; Global Biodiversity Information Facility: http://www.gbif.org/; NCBI Taxonomic Browser: http://www.ncbi.nlm.nih.gov/taxonomy; and regional and local faunas and floras)
Links to Wikipedia articles, using QRpedia to deliver content in the user’s preferred language.
Morphological databases (e.g., Morphbank: http://www.morphbank.net/, Paldat: http://www.paldat.org/)
Online maps or geographic gazetteers (e.g., Google Maps: https://maps.google.com/, Google Earth: http://www.google.com/earth, Mapquest: http://www.mapquest.com/, Yahoo! Maps: http://maps.yahoo.com/; Geographic Names Information System: http://geonames.usgs.gov/; GeoNames: http://www.geonames.org/; GEOnet Names Server: http://earth-info.nga.mil/gns/html/). See example in Fig. 4.
Links to protologs and libraries (e.g., Biodiversity Heritage Library: http://www.biodiversitylibrary.org; JSTOR Plant Science: http://about.jstor.org/global-plants)
Phylogenies (e.g., Tree of Life: http://tolweb.org/tree/, TreeBASE web: http://treebase.org/)
Links to funding agencies (e.g., projects funded using national and international resources could display that information)

## Final remarks

Are the QR codes going to be outdated soon? Are they going to be replaced by another 2D code? Similar questions were asked in the '60s concerning barcodes, and five decades later we are still using them. While there is no immediate answer to these questions, it must be pointed out that QR codes have rapidly inundated vast sectors of the industry. Long-term permanence of QR codes depends largely on popularity, and not even the High Capacity Color Barcodes (HCCB) released by Microsoft in 2007 acquired similar popularity in such a relatively short time.

Small institutions with tight budgets can still implement this technology, without having to spend preposterous amounts of money. If there is no Wi-Fi, users can still connect to the Internet through their phone networks. If there are no phone networks available, devices can be used to record the information contained in QR codes as text (e.g. label information from large batches of specimens).

One concern that has been expressed about QR codes is the potential presence of malicious code either in the codes themselves or on the websites they link to. Most of the scanning applications now have URL safety check services to detect malicious content. Recommendations for countering this problem are mentioned in Table 1.

Scanning QR codes using smartphones, iPods and digital tablets has become a common practice and there are more than 25 applications for doing this, most of them free of charge (see Appendix 2). QR codes are quickly penetrating the mainstream and represent an opportunity to facilitate access to specimen information. We already have a critical mass of the population with devices capable of using this technology, and in the near future people may be pointing their phones and tables to QR codes on natural history specimens to obtain more information.

## Appendix 1

List of URLs for QR Code generators. (doi: 10.3897/phytokeys.25.5175.app1) File format: Adobe PDF file (PDF).

## Appendix 2

List of URLs for QR Code scanning applications. (doi: 10.3897/phytokeys.25.5175.app2) File format: Adobe PDF file (PDF).
