# Supplementary material for: Utility of QR codes in biological collections
Source: PhytoKeys. 2013 Jul 17;(25):21–34. doi: 10.3897/phytokeys.25.5175 (PMC3819127; doi:10.3897/phytokeys.25.5175)
Supplement: Supplementary file 1 — List of URLs for QR Code generators. (doi: 10.3897/phytokeys.25.5175.app1) File format: Adobe PDF file (PDF). [file PhytoKeys-025-021-s001.pdf]

Appendix 1: List of URLs for QR Code generators

Advanced Telecom	http://www.atsqrcodes.com/free-qr-code-generator
AT&T Create-A-Code	http://www.createacode.att.mobiletag.com
AzonMobile*	http://www.azonmobile.com/en/qrcode-generator
Beautiful QR Codes	http://beautifulqrcodes.com
BeeTagg	http://www.beetagg.com
BeQRious	http://beqrious.com/qr-code-generator
BosqWeb QR Code generator*	http://www.bosqweb.net/en/QR-code-generator
CodeCreator	http://www.codecreator.org
ConnectMe QR	http://www.connectmeqr.com
CSouza QR Code Generator	http://qrcode.csouza.com
Delivr QR Code Generator*	http://delivr.com/qr-code-generator
FancyQRCode	http://www.fancyqrcode.com
GoQR.me*	http://goqr.me
HelloQR	http://helloqr.com
iCandy	http://icandy.ricohinnovations.com/rocket2
i-Nigma	http://www.i-nigma.com/CreateBarcodes.html
ItsMyURLs	http://itsmyurls.com
iTunes QR Pro	https://itunes.apple.com/us/app/id436732207?mt=8
JumpScan QR Code	http://jumpscan.com
Kaywa QR Code	http://qrcode.kaywa.com
Kerem Erkan QR Code Generator*	http://keremerkan.net/qr-code-and-2d-code-generator
Likify	http://www.likify.net
Mobilefish online QR Code generator*	http://www.mobilefish.com/services/qrcode/qrcode.php
myQR	http://myqr.co
Patryk QR Code Generator	http://freedockstar.com/qrcode
QR Stuff	http://www.qrstuff.com
QR.to	http://qr.to/en
QR4 Batch Generation	http://blog.qr4.nl/Batch-QR-Code.aspx
QReate & Track	http://qreateandtrack.com
QRExplore Bulk QR Code Generator*	http://www.qrexplore.com/generate
QRinkle	http://qrinkle.com
QRMobilize	http://qrmobilize.com
QRmyLife*	http://qrmylife.com
QRpedia*	http://qrpedia.org
QuickMark*	http://www.quickmark.com.tw/En/qrcode-datamatrix-generator
Raco QR Code Barcode Generator*	http://www.racoindustries.com/barcodegenerator/2d/qr-code.aspx
Response House	http://www.responsehouse.com
Small QR	http://www.qurify.com/en
SnapMaze QR Code Generator	http://www.snapmaze.com
Social QR Code	http://www.socialqrcode.com
SparqCode	http://www.sparqcode.com/static/maestro
Tag.cx	http://tag.cx
Tagginn	https://www.tagginn.com
Tago	http://tagomobile.com
TapCodes	http://www.tapcodes.com
uQR.me	http://uqr.me
ZXing Project QR Code Generator*	http://zxing.appspot.com/generator

* Recommended websites: these websites allow customizations such as choosing security level, type of content, output format, batch generation, and other functionalities.
