# Supplementary material for: Utility of QR codes in biological collections
Source: PhytoKeys. 2013 Jul 17;(25):21–34. doi: 10.3897/phytokeys.25.5175 (PMC3819127; doi:10.3897/phytokeys.25.5175)
Supplement: Supplementary file 2 — List of URLs for QR Code scanning applications. (doi: 10.3897/phytokeys.25.5175.app2) File format: Adobe PDF file (PDF). [file PhytoKeys-025-021-s002.pdf]

*Appendix 2: List of URLs for QR Code scanning applications*

| | |
|---|---|
| 2DSense | http://www.2dsense.com |
| Apodidae | http://www.apodidae.com/barcode-assistant |
| Bakodo | http://bako.do/ |
| AT&T Barcode Scanner | https://www.wireless.att.com//businesscenter/solutions/mobile-marketing/products/mobile-barcode-service.jsp |
| Barcode Scanner* | https://code.google.com/p/zxing/ |
| BeeTagg | http://www.beetagg.com/en |
| Blackberry QR Code Scanner™ Pro – Free | http://appworld.blackberry.com/webstore/content/13962/?lang=en |
| Code Muncher | http://www.codemuncher.com |
| i-Nigma* | http://www.i-nigma.com/Downloadi-nigmaReader.html |
| Kaywa | http://www.kaywa.com |
| Lynkee | http://www.lynkee.com |
| Mobiletag | http://www.mobiletag.com |
| NeoReader | http://www.neoreader.com |
| Okotag | https://play.google.com/store/apps/details?id=com.jaxo.android.okotag&hl=en |
| Optiscan | http://www.optiscan.com |
| QR app | http://qrapp.com |
| QR Code Scaning | http://www.qrcodescanning.com |
| QR Droid* | http://q.qr.ai |
| QR Pal | http://qrpal.com |
| Qrafter* | http://keremerkan.net/downloads |
| QRdeCODE | http://www.denso-wave.com/en/adcd/QRdeCODE/index.html |
| QRReader | http://www.tapmedia.co.uk/more-apps.htm |
| QuickMark* | http://www.quickmark.cn |
| Quick Scan | http://www.ihandysoft.com/apps.html |
| RedLaser* | http://redlaser.com |
| Scan* | http://scan.me |
| ScanLife* | http://www.scanlife.com |
| Tapmedia | http://www.tapmedia.co.uk |
| SemaCode QR Code Reader | http://semacode.com |
| ShopSavvy | http://shopsavvy.mobi |
| SnapMaze | http://www.snapmaze.com |
| UpCode | http://www.upcode.fi/mobile/pc_download.asp?language=1 |
| Zapper Scanner* | http://z.qr.ai |
| Zxing Barcode Scanner | https://play.google.com/store/apps/details?id=com.google.zxing.client.android |

* Recommended scanners: available in numerous languages, these applications read most of the versions of QR codes, and have features such as zooming, light, batch reading, saving text, security verification and others.
